# Integrated time-series transcriptomic and metabolomic analyses reveal different inflammatory and adaptive immune responses contributing to host resistance to PRRSV

**DOI:** 10.3389/fimmu.2022.960709

**Published:** 2022-10-20

**Authors:** Qingqing Wu, Yu Han, Xianmeng Wu, Yuan Wang, Qiuju Su, Yang Shen, Kaifeng Guan, Jennifer J. Michal, Zhihua Jiang, Bang Liu, Xiang Zhou

**Affiliations:** ^1^ Key Laboratory of Agricultural Animal Genetics, Breeding and Reproduction of Ministry of Education, College of Animal Science and Technology, Huazhong Agricultural University, Wuhan, China; ^2^ The Cooperative Innovation Center for Sustainable Pig Production, Wuhan, China; ^3^ Department of Animal Sciences and Center for Reproductive Biology, Washington State University, Pullman, WA, United States; ^4^ Hubei Hongshan Laboratory, Wuhan, China; ^5^ The Engineering Technology Research Center of Hubei Province Local Pig Breed Improvement, Huazhong Agricultural University, Wuhan, China

**Keywords:** PRRSV, Tongcheng pigs, disease resistance, transcriptome, metabolome, adaptive immunity, inflammatory response

## Abstract

Porcine reproductive and respiratory syndrome virus (PRRSV) is a highly contagious disease that affects the global pig industry. To understand mechanisms of susceptibility/resistance to PRRSV, this study profiled the time-serial white blood cells transcriptomic and serum metabolomic responses to PRRSV in piglets from a crossbred population of PRRSV-resistant Tongcheng pigs and PRRSV-susceptible Large White pigs. Gene set enrichment analysis (GSEA) illustrated that PRRSV infection up-regulated the expression levels of marker genes of dendritic cells, monocytes and neutrophils and inflammatory response, but down-regulated T cells, B cells and NK cells markers. CIBERSORT analysis confirmed the higher T cells proportion in resistant pigs during PRRSV infection. Resistant pigs showed a significantly higher level of T cell activation and lower expression levels of monocyte surface signatures post infection than susceptible pigs, corresponding to more severe suppression of T cell immunity and inflammatory response in susceptible pigs. Differentially expressed genes between resistant/susceptible pigs during the course of infection were significantly enriched in oxidative stress, innate immunity and humoral immunity, cell cycle, biotic stimulated cellular response, wounding response and behavior related pathways. Fourteen of these genes were distributed in 5 different QTL regions associated with PRRSV-related traits. Chemokine CXCL10 levels post PRRSV infection were differentially expressed between resistant pigs and susceptible pigs and can be a promising marker for susceptibility/resistance to PRRSV. Furthermore, the metabolomics dataset indicated differences in amino acid pathways and lipid metabolism between pre-infection/post-infection and resistant/susceptible pigs. The majority of metabolites levels were also down-regulated after PRRSV infection and were significantly positively correlated to the expression levels of marker genes in adaptive immune response. The integration of transcriptome and metabolome revealed concerted molecular events triggered by the infection, notably involving inflammatory response, adaptive immunity and G protein-coupled receptor downstream signaling. This study has increased our knowledge of the immune response differences induced by PRRSV infection and susceptibility differences at the transcriptomic and metabolomic levels, providing the basis for the PRRSV resistance mechanism and effective PRRS control.

## Introduction

Porcine reproductive and respiratory syndrome (PRRS), caused by PRRS virus (PRRSV), is a widespread viral swine disease that causes reproductive failure in sows and respiratory disease in pigs of any age (1). Swine production in the US, China and other countries all over the world suffer huge annual economic losses from PRRSV infections (2). PRRSV is characterized by high variability, persistent infections, immunosuppression, delayed appearance and low levels of neutralizing antibody, and antibody-dependent enhancement (2–4). Development of killed and modified-live PRRSV vaccines have failed to provide adequate protection against heterologous PRRSV strains (5–7). Therefore, the improvement of host resistance to PRRS may provide a more effective approach for PRRS control (8).

Previous studies clearly showed that genetic differences play an important role in susceptibility/resistance to PRRSV. Susceptibility of monocyte-derived macrophages to PRRSV were divergent among diverse commercial lines of pigs and greater PRRSV-induced lung lesions were reported in Large White (LW) pigs (9). The replication of PRRSV in porcine alveolar macrophages (PAMs) from Landrace pigs was significantly slower compared to PAMs from LW and Pietrain pigs (10). Interestingly, Chinese native breeds such as Tongcheng (TC) (11–13), Dingyuan (14) and Tibetan pigs (15) exhibit differential susceptibility to natural infections of PRRSV with mild lesion in lungs and low infection rate. These findings suggest that resistance to PRRSV infection is heritable. The host resistance to PRRSV is estimated by a combination of factors, such as viral loads, weight gains and regulation of innate and adaptive immune responses (8). A genome-wide association study revealed a major QTL associated with host response to PRRSV and moderate heritability of viral load and weight gain in crossbred pigs, impacting the severity and progression of disease (16). Recently, pigs with low tonsil PRRS viral levels were phenotypically related to earlier and faster serum virus clearance (17). Furthermore, recent studies discovered that polymorphisms in the genes *GBP5*, *CD163* and SNP rs80800372 (WUR) were associated with susceptibility/resistance to PRRSV (18–20).

Because the PRRSV can evade the porcine innate immune response, the development of an adaptive immune response may play a crucial role in virus clearance (21). At the early stage of infection, PRRSV induces a sharp decrease in white blood cell counts, lymphocytes and monocytes in piglets (2, 22–24). PRRSV-related lymphopenia mainly marked by changes in the subpopulations of T-lymphocytes, results in a weak and delayed adaptive immune response (25). Differences in T cell responses have been documented in pigs that have succumbed to or survived PRRSV infection (23). Moreover, T cell responses as measured by the number of virus-specific IFN-γ T cells have a striking association with the reduction of PRRSV viremia (26, 27). Thus, PRRS vaccines that target adaptive immune mechanisms and prevent virus-driven immunosuppression may be the most effective approach to preventing PRRSV infection (28, 29).

The integration of transcriptome and metabolome gives a comprehensive insight into understanding immune responses to viral infections like PRRSV (30). Several studies have further explored adaptive immune response differences to PRRSV infection using the RNA-Seq technique. The blood transcriptomes of pregnant gilts infected with PRRSV with low fetal mortality rates exhibited greater T cell activation than gilts with high fetal mortality rates (31). Interestingly, more active adaptive immune responses in lung transcriptomes were observed than in lymphoid organs of pigs infected with PRRSV (32). Dong et al. studied the tonsil transcriptome of PRRSV infected pigs and found that pigs with high tonsil virus levels potentially trigger stronger immune responses (33).

Additionally, metabolites also participate in the regulation and signal transduction of immune cells upon viral infection and inflammation response (34–36). Recent studies have suggested metabolic changes in host responses of pigs following Swine Fever Virus (37), Mycoplasma hyopneumoniae (38) and PRRSV (39) infections. In a recent study, alpha-AAA, kynurenine and lysoPCs were identified as potential metabolomic markers of PRRSV fetal susceptibility (39).

In a previous study, TC pigs had stronger resistance, less severe symptoms, lower viral load levels, and stable leukocyte counts compared to LW pigs during early PRRSV infection (12). Large genetic differences between TC and LW pigs have limited the identification of important genes associated with PRRSV susceptibility. Therefore, we constructed a crossbred population by crossing PRRSV-resistant TC pigs and PRRSV-susceptible LW pigs to the ninth generation. After several generations of recombination, the population generates a rich genetic resource with large phenotypic diversity suitable for studying the genetic architecture of disease resistance to PRRSV infection (40). Previous PRRSV artificial infection experiments in the crossbred population suggest significantly different immune responses between pigs who did or did not survive the infection, especially in serum viral load and lymphocyte percentage (41, 42). Our objective was to decipher the mechanisms of immune response differences for the susceptibility/resistance to PRRSV infection. In this study, we performed an integrated analysis of the white blood cells transcriptome and serum metabolome of infected piglets from the crossbred population described above at serial timepoints. Differentially expressed genes (DEGs) and metabolites participating in immune responses were identified to characterize the dynamic immune response differences between the resistant and susceptible pigs.

## Materials and methods

### Animal experiment and blood sampling

**Figure 1:**
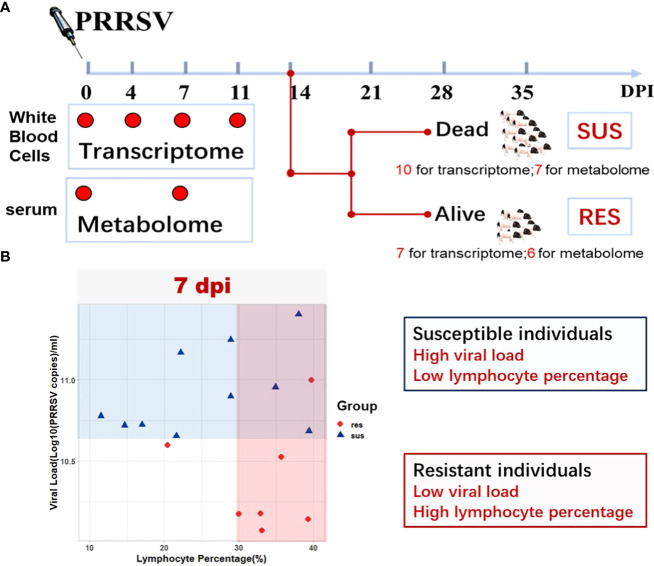
Study Overview. **(A)** Schematic representation of the experimental design in terms of sample types, target tissues, and sampling time points (0 dpi, 4 dpi, 7 dpi, 11 dpi) after PRRSV infection. **(B)** Serum viral loads and peripheral blood lymphocyte percentages in susceptible and resistant pigs.

In order to decipher the mechanism of disease resistance to PRRSV infection, we constructed a crossbred population of PRRSV-resistant TC pigs and PRRSV-susceptible LW pigs to the ninth generation. Seventy-four healthy weaned piglets with average weight of 15 kg were selected from this population. Piglets received a 2 ml intramuscular and 1 ml intranasal injection of the PRRSV strain WUH3 (virus dose: 10^5^ TCID_50_/ml) on day 0. Blood samples were collected in anticoagulation tube with EDTA-K2 at 0, 4, 7 and 11 days post infection (dpi) as illustrated in the diagram (**Figure 1A**). The white blood cells were separated from fresh blood by Red Blood Cell Lysis Buffer (Solarbio, China) and resuspended with RNAiso Plus reagent (Takara, Japan) for total RNA extraction. The serum samples were separated from the clot by centrifuging at 1000 g for 10 minutes in a refrigerated centrifuge (Eppendorf, Germany). White blood cell samples and serum samples were stored at -80℃. The determination method of serum viral loads by absolute quantitative RT-PCR assay was the same as previously described (12). The lymphocyte percentages in peripheral blood were detected by BC-2800VET Auto Hematology Analyzer (Mindray, China). All animal procedures were approved by the Ethical Committee for Animal Experiments at Huazhong Agricultural University, Wuhan, China. The animal experiments were performed at the Laboratory Animal Center of Huazhong Agricultural University (Animal experiment approval ID Number: HZAUSW-2017-005).

### RNA preparation and RNA-Seq

Sixty white blood cell samples from a total of 17 pigs at four timepoints (0 dpi, 4 dpi,7 dpi, 11 dpi) were used for RNA-seq (**Figure 1A**). Among them, 8 pigs died of PRRSV after 8 dpi. Detailed information on the experimental design is provided in **Figure 1A**. Total RNA was isolated from white blood cells using the RNAiso Plus reagent (Takara, Japan) according to the manufacturer’s instructions. RNA quality was assessed on an Agilent 2100 Bioanalyzer (Agilent Technologies, USA). Then the mRNAs were enriched by oligo(dT) magnetic beads and fragmented with fragmentation buffer. Six-base random hexamers were used to synthesize the first cDNA strand, and then second-strand cDNA fragments were synthesized by DNA polymerase I, RNase H, dNTP and buffer. The cDNA fragments were purified with QiaQuick PCR extraction kit (Qiagen, Germany), end repaired, poly(A) added, ligated to Illumina sequencing adapters and then size selected to construct sequencing libraries. All libraries were sequenced on Illumina NovaSeq 6000 platform with a 150-bp paired-end module at Berry Genomics Company (Beijing, China). Clean reads were obtained by removing reads containing adapter or poly-N and low-quality reads from raw reads. Clean reads were aligned against ENSEMBL Suscrofa11.1 with TopHat v2.0.9 (available online: https://github.com/infphilo/tophat) and bowtie v2.1.0 (available online: http://bowtie-bio.sourceforge.net/index.shtml).

### Transcriptome data analysis

The raw sequence count data were normalized with the edgeR R package (43) using the “TMM” method and genes were excluded when all samples had raw counts of ≤ 10 to reduce statistical bias. DEGs were identified using the limma R package (44). The resulting p-values were adjusted using Benjamini and Hochberg’s approach for controlling the false discovery rate. Genes with |log2(FoldChange)| > 1 and adjusted p-value < 0.05 found by limma were assigned as differentially expressed. Gene set enrichment analysis (GSEA) was performed using the java GSEA software (45). Blood Transcription Modules (BTMs) were used as gene sets and module activity scores were taken as the mean value of member genes (46). Significant enrichment was determined by a false discovery rate (FDR) < 0.05 and normalized enrichment score (NES) >1.

### Cell composition analysis

The RNA-Seq dataset consisting of sixty white blood cell samples was analyzed by CIBERSORT (47) R package to quantify immune cell compositions. The gene expression dataset was run using the LM22 signature and 100 permutations. The proportions of 22 cell types in each sample were estimated and their differences between resistant pigs and susceptible pigs were tested by Wilcoxon’s test. Statistical significance was defined as a p-value less than 0.10.

### Functional analysis of differential expressed genes

Three RNA-seq time course tools (ImpulseDE2, splineTC and maSigPro) based on different core methods were used for identifying the DEGs between susceptible and resistant pigs (48–50). The DEGs between susceptible and resistant pigs were clustered using a hierarchical clustering method with the clustering function of the maSigPro R package. Functional enrichment analyses were performed by clusterProfiler R package, using biological process terms in the Gene Ontology (GO) database for the genes in each cluster (51). The top eight terms in the enrichment results for each cluster were used for visualization. The positions of DEGs were matched to quantitative trait loci (QTL) regions for PRRSV related traits in the pig QTL database (http://www.animalgenome.org/cgi-bin/QTLdb/SS/index).

### Quantitative PCR validation of differential expressed genes

Total RNA from three resistant pigs and three susceptible pigs at three different timepoints (0, 4 and 7 dpi) were used for quantitative PCR (qPCR) validation. Samples were reverse transcribed into cDNA using the PrimeScript™ RT reagent kit with gDNA Eraser (TaKaRa, Japan) according to the manufacturer’s instructions. The qPCR reactions were performed by SYBR Green method using the CFX384 Touch Real-Time PCR Detection System (Bio-Rad, USA). The 10 μL qPCR reactions comprised 5 μL 2× TB Green *Premix Ex Taq* II (TaKaRa, Japan), 0.2 μM primers designed for target genes, and 1 μL of cDNA sample. Every assay for target genes included no-template controls and every sample was analyzed in triplicate. The thermocycler program consisted of an initial hot start cycle at 95 °C for 30 sec, followed by 40 cycles at 95 °C for 5 sec and 60 °C for 30 sec with melting curve analysis. Relative expression levels were normalized to the expression level of reference gene RPS18 (52) and calculated using the 2^-ΔΔCt^ method. qPCR results were presented as fold changes relative to the expression level of each sample at 0 dpi. The primers used for qPCR are shown in **Supplementary Table 3**.

### Metabolomic profile using liquid chromatography-mass spectrometry

Twenty-sixty serum samples collected from 13 pigs at 0 dpi (pre-infection) and 7 dpi (post-infection) were used for liquid chromatography-mass spectrometry (LC-MS) analyses (**Figure 1A**). Methanol (precooled at -2 0°C) was added to thawed samples and centrifuged for 10 min at 12,000 rpm at 4°C. A 20 µl aliquot from each sample was used for quality control assessment and the remaining sample was used for LC-MS detection. Chromatographic separation was accomplished in a Thermo Ultimate 3000 system equipped with an ACQUITY UPLC^®^ HSS T3 (150×2.1 mm, 1.8 µm, Waters) column maintained at 40°C. The ESI-MSn experiments were executed on the Thermo Q Exactive mass spectrometer with the spray voltage of 3.8 kV and -2.5 kV in positive and negative modes, respectively. The original dataset was used for peak picking, peak alignment and peak annotation by XCMS and CAMERA. Briefly, the UPLC/MS/MS product ion spectra were annotated using the Human Metabolome Database (http://www.hmdb.ca), Metlin (http://metlin.scripps.edu), massbank (http://www.massbank.jp/), Lipid Maps (http://www.lipidmaps.org), mzCloud (https://www.mzcloud.org), and BioNovoGene Company (http://www.bionovogene.com) standard database and Kyoto Encyclopedia of Genes and Genomes databases (KEGG, http://www.genome.jp/kegg/). The resulting peak intensity table was exported for comprehensive statistical and functional analyses.

### Metabolomics data analyses

The peak intensity table consists of samples in columns and metabolic features in rows. The peak intensity matrix was normalized by log2 transformation, followed for partial least squares discriminant analysis (PLS-DA) using MetaboAnalyst 5.0 (53). Pairwise comparisons were conducted using the Wilcoxon’s test. The differentially expressed (DE) metabolites were filtered with the following cut-off: |log2(FoldChange)| > log2(1.5) and p-value < 0.05. The functional enrichment pathways were identified by MetaboAnalyst 5.0 using databases KEGG. Besides pairwise comparisons, we also performed one-way analysis of variance (ANOVA) to identify the DE metabolites (FDR < 0.10). DE metabolites were clustered using a hierarchical clustering method in R.

### Joint analyses of transcriptomics and metabolomics data

The transcriptomics and metabolomics datasets were collapsed into their respective clusters by unsupervised hierarchical clustering. The cluster scores were taken as the mean value of member genes or metabolites. The BTM clusters for analyses were those gene sets from significantly enriched BTMs at 7 dpi in the GSEA results. Pearson’s correlation coefficients between the scores of transcriptomic clusters and metabolomic clusters were calculated by R. Associations between transcriptomic clusters (or genes) and metabolomic clusters (or metabolites) were estimated by general linear regression (GLM). The integrated enrichment pathway analyses of DEGs and DE metabolites were performed by IMPaLA webtools (http://impala.molgen.mpg.de/). The resulting networks were visualized using Cytoscape 3.8.2 (http://cytoscape.org).

## Results

### Overview of transcriptome data reveal different immune response to PRRSV between resistant pigs and susceptible pigs

**Figure 2:**
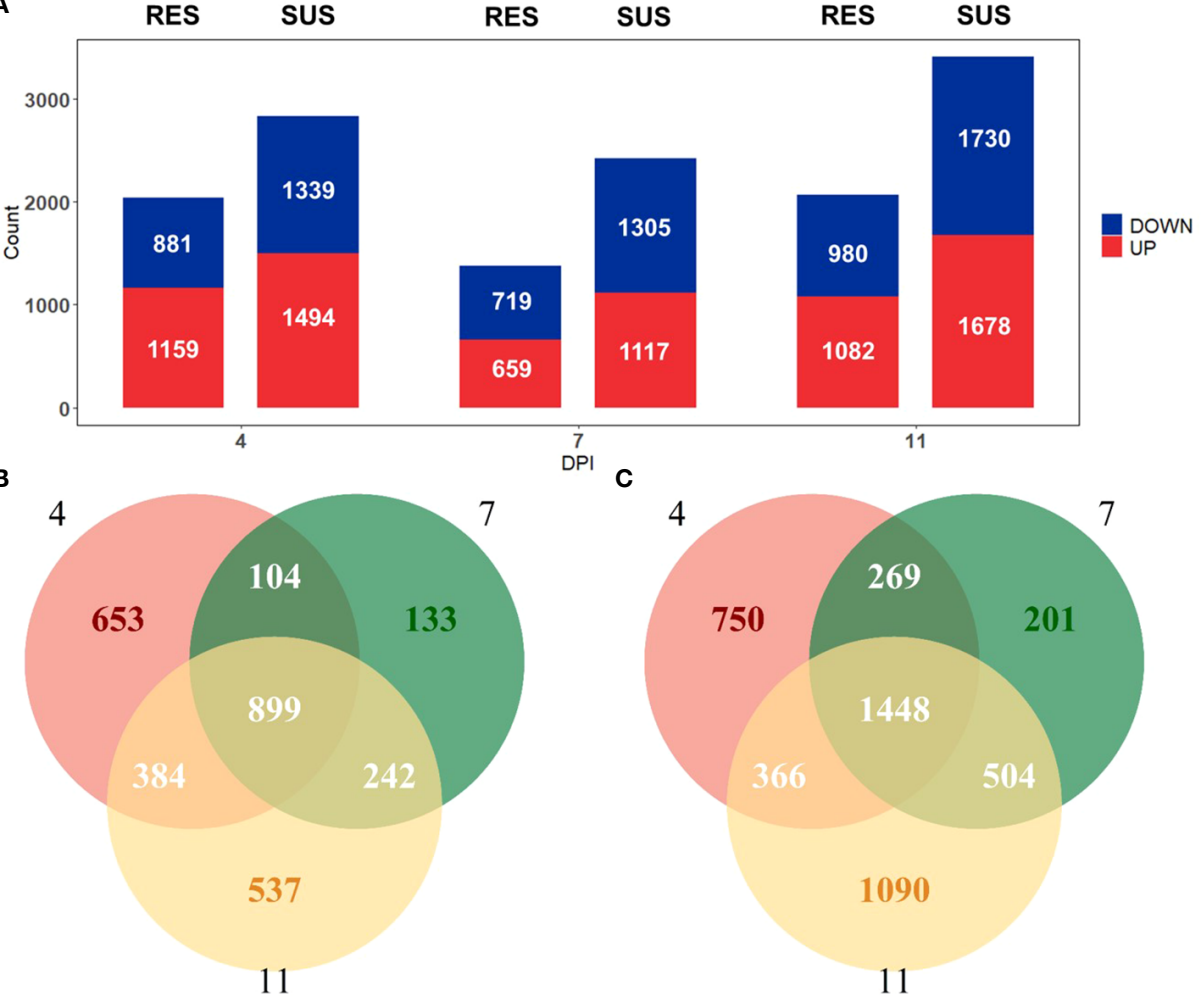
**(A)** Number of genes differentially expressed (|log2(FoldChange)| > 1 and adjusted p-value < 0.05) relative to day 0 in resistant (left) and susceptible (right) pigs on days 4, 7 and 11 post-infection. **(B)** Venn diagram comparing identified DEGs in resistant pigs. **(C)** Venn diagram comparing identified DEGs in susceptible pigs.

To identify how the peripheral blood cell transcriptome is influenced by PRRSV infection, we performed challenge trials with 74 pigs that were infected with PRRSV strain WUH3. Based on survival time, serum viral loads and lymphocyte percentage post-PRRSV infection, we classified 17 pigs into susceptible (n = 10) and resistant (n = 7) groups (**Supplementary Table 1**). Susceptible pigs were characterized by high viral loads and low lymphocyte percentage at 7 dpi and death before 14 dpi, while resistant pigs had low viral loads and high lymphocyte percentages at 7 dpi and survived past 14 dpi (**Figures 1A, B**). Paired-end sequences from 60 white blood cell samples from 17 pigs collected at 0, 4, 7 and 11 dpi were generated. Approximately 83.51% of the 2.5 billion sequenced reads (an average of 41.3 million paired-end reads per sample) were mapped to the pig reference genome *Sscrofa11.1* (**Supplementary Table 2**). Differential expression analyses were respectively performed in susceptible-, resistant- and all pigs by comparing blood transcriptome datasets at 4,7, and 11 dpi with 0 dpi (**Figure 2A**). We identified 2952 DEGs in resistant pigs and 4628 DEGs in susceptible pigs in response to PRRSV infection for at least one of the collection times (adjusted p-value < 0.05 and |log2(FoldChange)| > 1). The overlapping DEGs are shown in Venn diagrams (**Figures 2B, C**). There were 30.1% (899/2952) and 31.3% (1448/4628) DEGs in common among the three collection timepoints in resistant and susceptible pigs, respectively (**Figures 2B, C**). Moreover, the susceptible pigs showed a relatively modest increase in the number of DEGs at all time points compared to the resistant pigs. Our results demonstrate that PRRSV infection induces a greater number of DEGs in susceptible pigs than resistant pigs.

**Figure 3:**
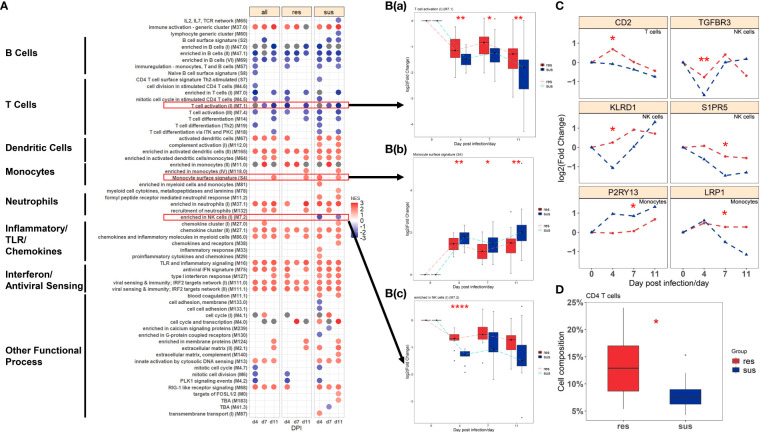
**(A)** BTMs significantly enriched (FDR < 0.05 & NES >1) in all (left), resistant (center) and susceptible (right) pigs post infection by GSEA; Blue to red scale indicates negative or positive associations based on normalized enrichment scores (NES). **(B)** Temporal expression patterns of modules with significant differences between resistant (red) and susceptible (blue) pigs: (a) M7.1: T cell activation(II); (b) S4: Monocyte surface signatures; (c) M7.2: enriched in NK cells(I). **(C)** Temporal expression patterns of genes with significant differences between resistant (red) and susceptible (blue) pigs. Significance levels are shown as *, p-value < 0.05; **, p-value < 0.01; ****, p-value < 0.0001. **(D)** The proportion of CD4 T cells between resistant (red) and susceptible (blue) pigs.

Gene set enrichment analysis (GSEA) was employed to identify the transcriptional pathways related to PRRSV infection in all pigs, resistant pigs and susceptible pigs. BTMs with higher sensitivity of capturing immunological events from blood transcriptomics were used as enrichment gene sets. A summary of significantly enriched BTMs is shown in **Figure 3A** (FDR < 0.05 & NES >1). All three groups exhibited up-regulation of pathways related to activation of dendritic cells, monocytes and neutrophils and inflammatory/TLR/chemokines responses and down-regulation of pathways associated with activation of T cells, B cells and NK cells after PRRSV infection (**Figure 3A**). Notably, comparison of susceptible and resistant pigs revealed significant differences in three modules (p-value < 0.05, **Figure 3B**). The resistant pigs showed a higher level of T cell activation module (M7.1) at 4, 7, 11 dpi (**Figure 3B(a)**, **Supplementary Figure 1A**) and were highly enriched in the NK cells module (M7.2) at 4 dpi (**Figure 3B(c)**, **Supplementary Figure 1C**). In comparison, monocyte surface markers (S4) were more abundant in susceptible pigs at 4, 7, 11 dpi (**Figure 3B(b)**, **Supplementary Figure 1B**). The abundances of several T cell, NK cell and monocyte markers including *CD2*, *TGFBR3*, *KLRD1*, *LRP1* were significantly different between resistant and susceptible pigs (p-value < 0.05, **Figure 3C**). Expression levels of *CD2*, *P2RY13*, *KLRD1* and *LRP1* were also validated by qPCR (**Supplementary Figure 2**, **Supplementary Table 3**). Immune cell compositions were estimated by CIBERSORT analysis from the gene expression profiles (**Supplementary Figure 3**). Among 6 immune cell types, T cells were the most prevalent population in both groups at 0 dpi, while the proportions of lymphocytes (B cells and T cells) decreased and monocytes and macrophages were the most prevalent populations at 4 dpi. Significant differences were observed in the proportions of T cells (p-value = 0.055), dendritic cells (p-value = 0.043) and neutrophils (p-value = 0.088) at 4 dpi between resistant/susceptible pigs (**Supplementary Figure 3A).** Resistant pigs have higher average percentages among almost all T cell subpopulations and have significantly greater numbers of Tregs (**Supplementary Figure 3B**) and the proportion of sum of CD4 T cell subpopulations at dpi 4 (p-value < 0.10, **Figure 3D**). Overall, these data indicate that susceptible pigs developed a stronger T cell suppression and monocyte activation at 4, 7, 11 dpi, which may contribute to the lower lymphocyte percentage and severe symptoms after PRRSV infection (**Figure 1B**).

### Transcriptome profile reveals susceptible pigs have severe immune response to PRRSV

**Figure 4:**
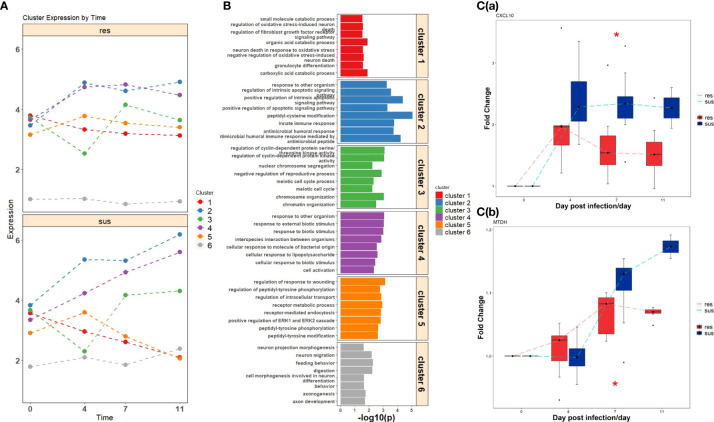
**(A)** Temporal expression patterns of clustered DEGs in resistant (top) and susceptible (bottom) pigs post infection. **(B)** Gene ontology enrichment analyses of DEGs within clusters. **(C)** The expression values of significant DEGs related to immune response between resistant (red) and susceptible (blue) pigs; (a) CXCL10, (b) MTDH. Significance level is shown by *, p-value < 0.05.

A total of 209 DEGs between susceptible and resistant pigs identified by time-course expression analyses were grouped into six gene sets by hierarchical clustering (**Figure 4A**, **Supplementary Figure 4**). These clusters displayed different temporal expression patterns between susceptible and resistant pigs (**Figure 4A**). The DEGs from these six clustered DEGs sets were subjected to GO enrichment analyses, revealing the top eight GO terms shown in **Figure 4B**. The DEGs within the 6 clusters were respectively enriched in oxidative stress (cluster 1), innate immunity and humoral immunity (cluster 2), cell cycle (cluster 3), biotic stimulated cellular response (cluster 4), wounding response (cluster 5) and behavior (cluster 6) related pathways. More specifically, abundances of *CXCL10* and *MTDH* were significantly differential between susceptible/resistant pigs at 7 dpi, and the susceptible pigs had higher fold changes than resistant pigs (**Figure 4C** and **Supplementary Figure 2**). Furthermore, we matched positions of DEGs to PRRSV-related QTLs. Fourteen DEGs, including *LMNA*, *PRPF3*, *ST18*, *CENPQ*, *KLF15*, *KIF7*, *CRISP3*, *TAC1* and *SHTN1* were distributed in 5 different QTL regions associated with PRRSV-related traits (**Supplementary Table 4**) (54–56).

### Overview of metabolome data reveal distinct metabolomic responses to PRRSV between resistant and susceptible pigs

**Figure 5:**
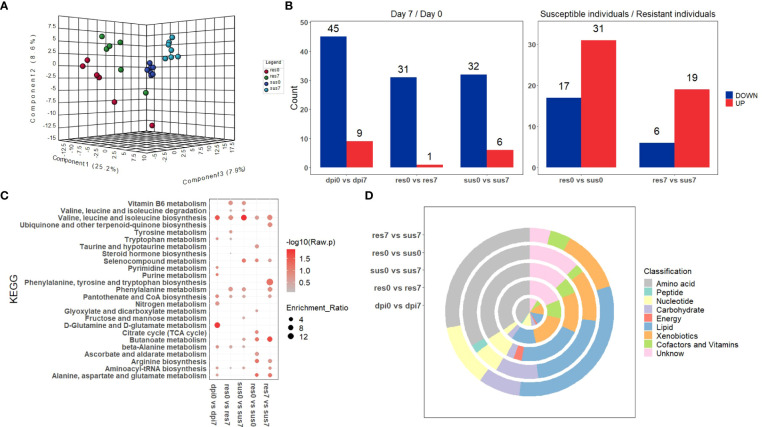
**(A)** PLS-DA of metabolite-intensity data in resistant and susceptible pigs on days 0 and 7 post-infection; **(B)** Number of metabolites differentially expressed (|log2(FoldChange)| > log2(1.5) & p-value < 0.05) in resistant and susceptible pigs on days 0 and 7 post-infection; **(C)** KEGG enrichment analysis of DE-metabolites within five groups; **(D)** The classification of significant DE-metabolites within five comparisons.

The samples were divided into four classes according to their susceptibility/resistance to PRRSV and their timepoints (0 dpi or 7 dpi), labeled as “sus0”, “sus7”, “res0”, “res7”. A final matrix (302 metabolites × 26 samples) was employed to build a PLS–DA classification model. The four groups (res0, res7, sus0, sus7) were distinct and the two resistant groups (res0 and res7) were distinguished from susceptible groups (sus0 and sus7) by three components (**Figure 5A**), indicating significant differences underlying host susceptibility to PRRSV infection. To investigate the metabolomic changes induced by PRRSV infection, the pre-infection (0 dpi) vs post-infection (7 dpi) comparisons were performed in three groups (all pigs, resistant pigs and susceptible pigs). The DE metabolites (|log2(FoldChange)| > log2(1.5) & p-value < 0.05) were mainly down-regulated after PRRSV infection in all three groups (**Figure 5B**). The KEGG enrichment analysis of those DE metabolites demonstrated that most significant pathways driven by PRRSV infection were in amino acid metabolism and synthesis, such as valine, leucine and isoleucine biosynthesis and D-glutamine and D-glutamate metabolism (**Figure 5C**). Moreover, the majority of DE metabolites were categorized in amino acid and lipid classifications (**Figure 5D**).

**Figure 6:**
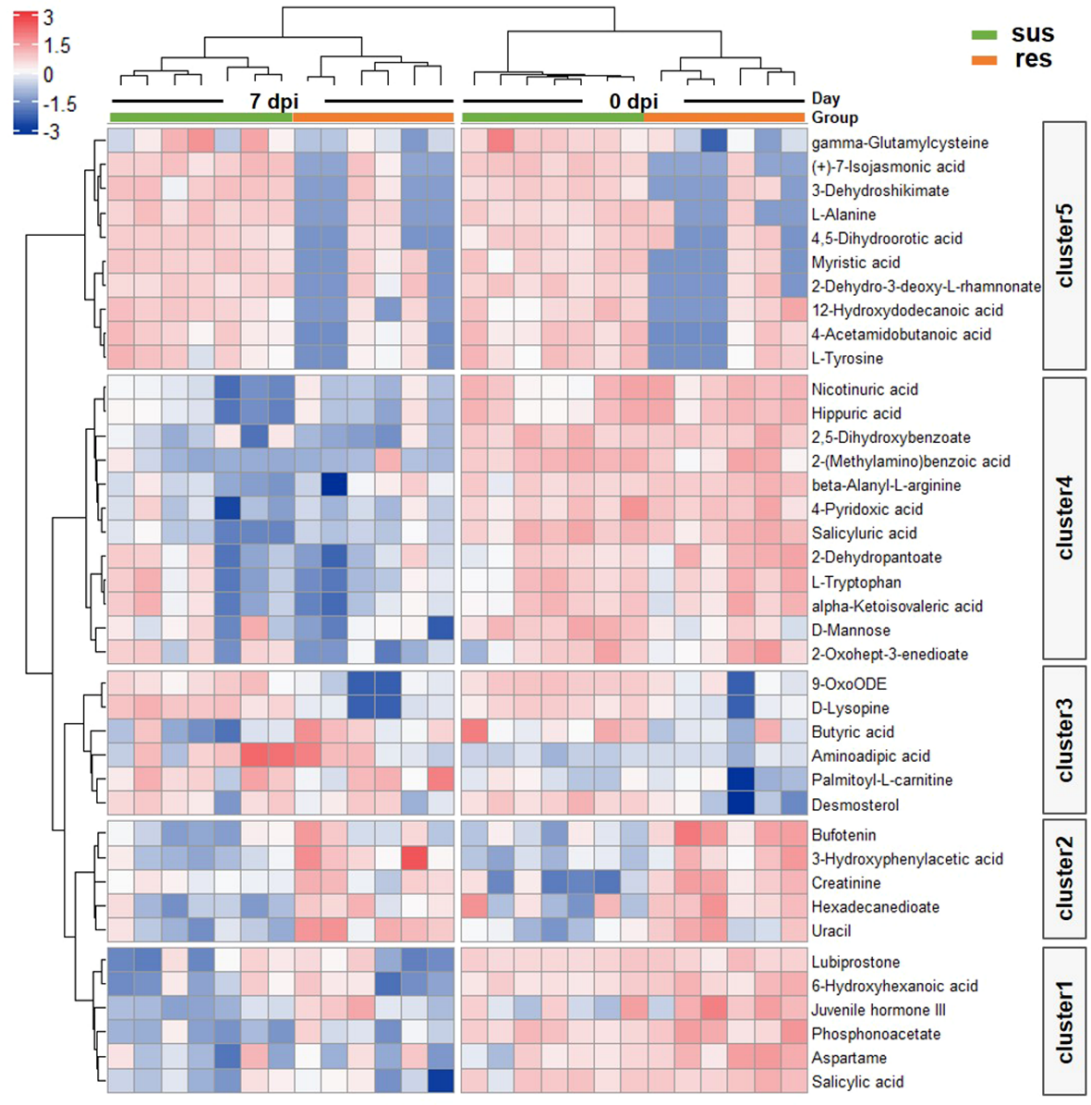
Heatmap of DE metabolites by one way ANOVA. Blue to red scale indicates negative or positive associations based on metabolites expression levels. Each column represents a sample, and each row represents a DE metabolite.

There was a total of 48 DE metabolites detected between the susceptible/resistant pigs at 0 dpi, while fewer numbers were seen at 7 dpi (**Figure 5B**). These results may indicate that the baseline differences were greater between the susceptible/resistant pigs. Notably, most of the DE metabolites were at higher levels in the susceptible pigs (**Figure 5B**). It appears that butanoate (short chain fatty acids) metabolism was the most significantly enriched pathway between susceptible/resistant pigs at 7 dpi (**Figure 5C**). A large proportion of lipids were significantly different between susceptible/resistant pigs at 7 dpi (**Figure 5D**). A one-way ANOVA analysis was performed to identify DE metabolites between the four groups (res0, res7, sus0, sus7). A total of 39 metabolites were significantly different (FDR < 0.1) and were classified into 5 clusters using unsupervised clustering (**Figure 6**). Obviously, the DE metabolites in cluster 1 and cluster 4 were induced by PRRSV infection, while cluster 2, cluster 3 and cluster 5 were responsible for the susceptibility differences to PRRSV (**Figure 6**, **Supplementary Table 5**).

### The integrative analysis of transcriptome and metabolome revealed DE metabolites positively correlated with DEGs in T cell and B cell signatures in response to PRRSV infection

**Figure 7:**
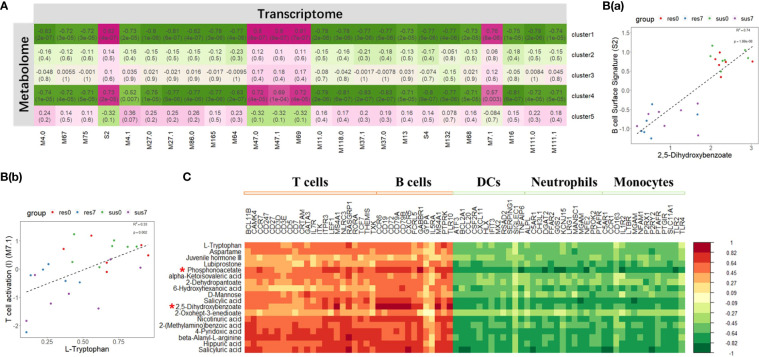
**(A)** Correlation between transcriptomic BTM clusters and metabolomic clusters; The magenta to green scale color indicates a positive to negative Pearson’s correlation coefficient, and coefficient values and the corresponding p-values were labeled on the boxes. **(B**(a)**)** Correlation between B cell surface signature (S2) module and 2,5-dihydroxybenzoate. **(B**(b)**)** Correlation between T cell activation (II) (M7.1) module and L-tryptophan. **(C)** Correlation between DE metabolites in cluster 1, 4 and marker genes in T cells, B cells, DCs, neutrophils and monocytes related modules; the horizontal axis represents DEGs, and the vertical axis represents DE metabolites. Top 2 significant metabolites were marked by "*".

The metabolomic clusters 1 and 4 were significantly correlated with all BTM clusters (**Figure 7A**). Specifically, they were positively correlated with the BTMs related to T cell and B cell activation, but negatively correlated with the BTMs related to DC, neutrophil and monocyte activation (**Figure 7A**). 2,5-dihydroxybenzoate, L-tryptophan, D-mannose, salicyluric acid and phosphonoacetate in clusters 1 and 4 were significantly correlated with those marker genes in BTMs (**Figure 7C**). Focusing on the metabolite levels, the intensity of 2,5-dihydroxybenzoate (2,5-DHBA) was significantly associated with the activity score of B cell surface signature (S2) module (R^2 =^ 0.74, p-value = 1.88×10^-8^) and L-tryptophan was significantly associated with the activity score of the T cell activation (M7.1) module (R^2 =^ 0.33, p-value = 0.002) (**Figure 7B**). The intensity of 2,5-DHBA and L-tryptophan were significantly down-regulated by PRRSV infection (p-value < 0.05) and associated with B cell and T cell activation modules.

### The integrative analysis of transcriptome and metabolome revealed that creatinine contributes to PRRSV-induced inflammation

**Figure 8:**
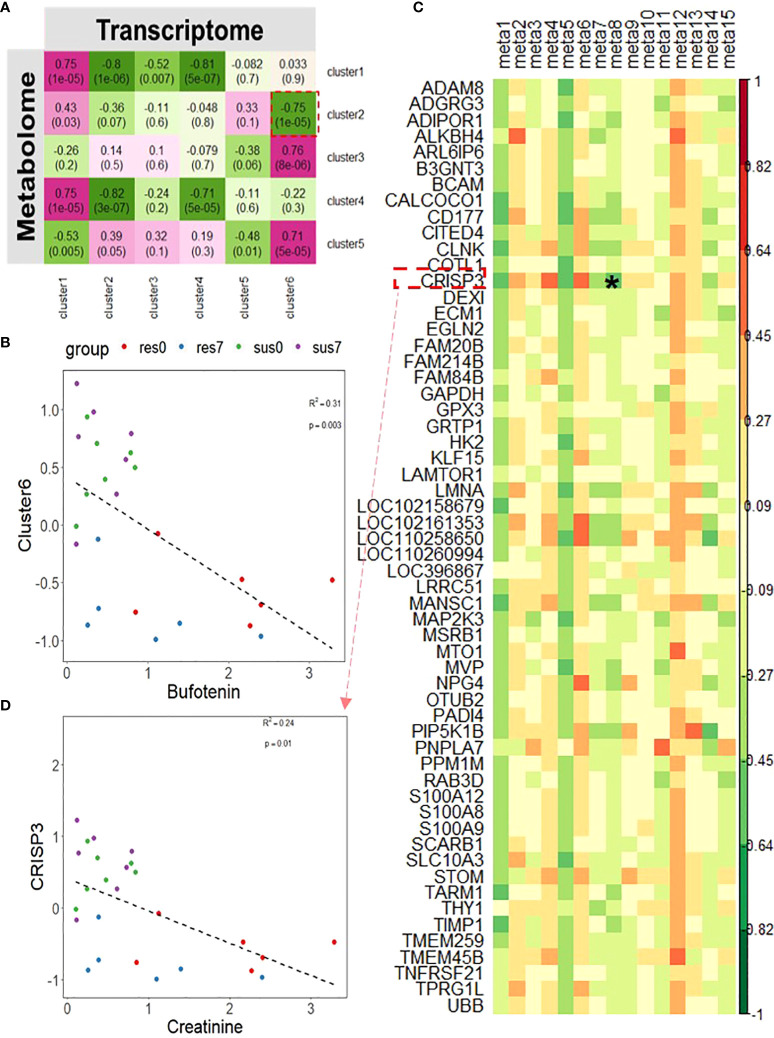
**(A)** Correlation between transcriptomic clusters of DEGs between resistant/susceptible pigs and metabolomic clusters; The magenta to green scale color indicates a positive to negative Pearson’s coefficient value, and coefficient values and the corresponding p-values are labeled on the boxes. **(B)** Correlation between bufotenin and transcriptomics cluster 6. **(C)** Correlation between DE metabolites in metabolomics cluster 2 and 5 and DEGs in transcriptomics cluster 2; the horizontal axis represents DE metabolites, and the vertical axis represents DEGs. Candidate combination of the metabolite and gene was marked by "*". **(D)** Correlation between creatinine and CRISP3. meta1~meta15 respectively represent metabolites in cluster 2 and 5: Bufotenin, (+)-7-Isojasmonic acid, 12-Hydroxydodecanoic acid, Myristic acid, Hexadecanedioate, L-Alanine, Uracil, Creatinine, 4,5-Dihydroorotic acid, 2-Dehydro-3-deoxy-L-rhamnonate, 4-Acetamidobutanoic acid,3-Dehydroshikimate, gamma-Glutamylcysteine, 3-Hydroxyphenylacetic acid, L-Tyrosine.

Using unsupervised hierarchical clustering, DEGs between resistant and susceptible pigs by time-course analyses were grouped into six clusters and DE metabolites were assembled into five clusters. We performed the correlation analysis between the six transcriptomic clusters and five metabolomic clusters and found that transcriptomic cluster 6 related to behavior (**Figure 4B**) was negatively correlated with the metabolomic cluster 2 (r = -0.75, p-value = 1×10^-5^) (**Figure 8A**). Particularly, bufotenin (member metabolite in cluster 2) was significantly associated with the scores of the transcriptomic cluster 6 (R^2 =^ 0.31, p-value = 0.003, **Figure 8B**), with significantly higher levels in resistant pigs regardless of PRRSV infection. Moreover, transcriptomic cluster 2 was significantly enriched with genes related to innate immune response and humoral immunity (**Figure 4B**). After correlation analysis of expression levels of member genes in transcriptomic cluster 2 with all member metabolites in metabolomic cluster 2 and cluster 5 (p-value < 0.05), we found that the gene *CRISP3*, which is located in the PRRSV susceptibility QTL (**Supplementary Table 4**) was significantly correlated with creatinine (R^2 =^ 0.24, p-value = 0.01, **Figures 8C, D**).

### Integrative pathway analysis revealed that DEGs and DE metabolites were significantly enriched in the GPCR downstream signaling pathway

**Figure 9:**
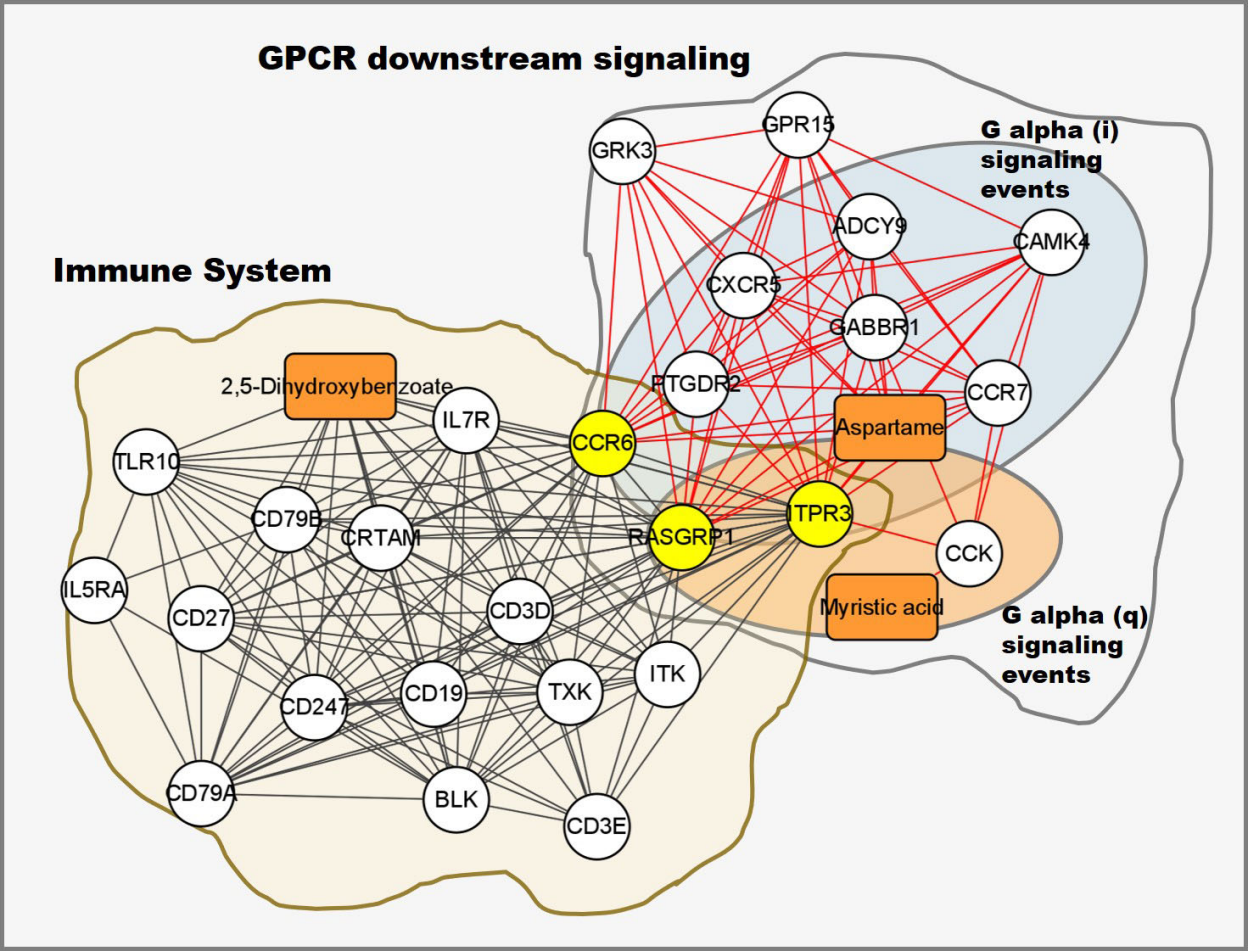
Integrated transcriptomic and metabolomic response network to PRRSV infection. Each node represents a significant correlation (|r| > 0.3 & p-value < 0.05), box represents metabolite, circle represents gene, the shared genes between immune system and GPCR downstream signaling pathways colored in yellow.

Integrated pathway enrichment analysis was performed using the total number of DEGs and DE metabolites. The most significantly enriched pathway was G protein-coupled receptor (GPCR) downstream signaling (p-value = 8.55×10^-5^). The sub-pathways of GPCR downstream signaling, including G alpha (i) signaling events, G alpha (q) signaling events and GPCR ligand binding, were significantly enriched as well (p-value < 0.05). DEGs and DE metabolites were also significantly enriched in the immune system pathway (p-value = 0.027). Genes *CCR6*, *RASGRP1* and *ITPR3* were in both the immune system pathway and the GPCR downstream signaling pathway (**Figure 9**). To further understand how PRRSV infection affects the two pathways, the expression levels of enriched DEGs and DE metabolites involved in both pathways were visualized by generating a heatmap, which showed that most components were down-regulated (**Supplementary Figure 5A**). Notably, susceptible pigs showed significantly higher levels of myristic acid than resistant pigs at both 0 and 7 dpi (**Supplementary Figure 5B**).

## Discussion

Because PRRSV vaccines provide limited protection against the disease, deciphering the mechanism underlying host resistance to PRRSV is crucial for effective PRRSV control. Using a resistant × susceptible crossbred pig population, we found significant immune response differences between pigs who died and those who survived the infection in the current PRRSV challenge study. Specifically, dead pigs had high viral loads and low lymphocyte percentages while pigs who survived the infection had low viral loads and high lymphocyte percentages at 7 dpi (**Figure 1B**). Recent studies have described that pigs with high levels of viremia have slower viral clearance and stronger immune responses than pigs with low levels of viremia (17, 33). PRRSV infection will induce a general decline in lymphocyte counts. Lymphopenia tends to be linked to a weak and delayed adaptive immune response (25). A similar phenomenon was found in humans infected with COVID-19 where the degree of lymphopenia predicts disease severity in COVID-19 patients (57). Antibody-mediated humoral immunity appears to provide minimal protection against viral infections. In comparison, cell-mediated immunity is essential to combat viral diseases such as COVID-19, PRRS and Marek’s disease, especially when antibody levels are low or declining (58–62). Therefore, viral loads and lymphocyte percentages may indicate the susceptibility of pigs to PRRSV infection.

The GSEA of the transcriptome dataset illustrated that PRRSV infection up-regulated genes involved in myeloid cells (dendritic cells, monocytes and neutrophils) related modules and inflammatory response modules, but down-regulated genes involved in lymphoid cells (T cells, B cells and NK cells) modules (**Figure 3A**). Notably, the majority of DE metabolites, such as 2,5-DHBA, L-tryptophan, D-mannose, salicyluric acid and phosphonoacetate, were down-regulated in response to PRRSV infection and were positively correlated to T cell and B cell marker genes (**Figure 7C**). L-Tryptophan and D-mannose appear to play important roles in T cell immunity (63–65).

Phosphonoacetate inhibited the growth of African swine fever virus in cultured swine monocytes (66). 2,5-DHBA suppresses siderophore synthesis *via* TLR signaling to regulate the innate immune response to bacterial infection (67). Increased alpha-aminoadipic acid and kynurenine levels were reported in PRRSV infected fetuses, especially in the fetuses with high viral load (39). In this study, aminoadipic acid levels were similarly increased in all infected pigs and L-kynurenine levels were increased in the susceptible pigs after PRRSV infection, but there were no significant differences between resistant and susceptible pigs (**Figure 6** and **Supplementary Figure 6**). Additionally, the majority of DE metabolites and DEGs enriched in the immune system pathway and GPCR downstream signaling pathways were down-regulated by PRRSV infection (**Figure 9**). The WUR SNP on chromosome 4 is a known marker of susceptibility/resistance to PRRSV in commercial pigs. Several studies reported that the unfavorable genotype AA animals had higher levels of viremia than AB and BB animals (16, 68, 69). A more recent study compared the transcriptomes of pigs infected with PRRSV with AB and AA WUR genotypes and found the GPCR pathway at 7 dpi was the most significant transcriptional difference and may contribute to the susceptibility differences to PRRS (70). In our data, the WUR genotypes of the infected piglets had no significant associations with viral loads and weight gain post PRRSV infection (p-value>0.05), but the most significantly enriched pathway was the GPCR pathway at 7 dpi. GPCR signaling is essential for the control of leukocyte migration patterns in immune responses, guiding T cell and B cell responses to eliminate or control pathogen invasions (71). CXCR5 and CCR7 are involved in the GPCR downstream signaling pathway and can regulate cell migration speeds. In fact, CCR7-deficient T cells have a 30%~50% reduction of T cell velocity *in vivo* (72). *RASGRP1* was shared by the immune system pathway and the GPCR downstream signaling pathway and was positively correlated with several DE metabolites (**Figure 7C**). In addition, the serum 2,5-DHBA levels were highly correlated with numerous genes in the immune response pathways (**Figure 9**). DEGs in the T cell and B cell modules and DE metabolites post PRRSV infection were generally down-regulated and positively correlated, indicating their potential cooperative roles in adaptive immune responses.

Interestingly, the expression profiles of resistant pigs indicate less suppression of T cell activation than susceptible pigs, corresponding to their higher lymphocyte percentage post PRRSV infection (**Figure 3B(a)**, **Supplementary Figure 1A**). Resistant pigs had a significant increase in the expression level of *CD2* (**Figure 3C**), which is generally up-regulated in activated T cells and memory T cells (73). Moreover, CIBERSORT analysis showed that resistant pigs had significantly higher numbers of T cells and CD4 T cell proportion at 4 dpi (**Figure 3D** and **Supplementary Figure 3**). A lower percentage of CD4 T cell counts in peripheral blood was associated with the disease severity and CD4+ counts were lower pigs that died compared to pigs that survived PRRSV infection in a previous study (23, 74). Several studies reported positive correlations between *CXCL10* and increased disease severity and risk of mortality in COVID-19 patients (60, 75, 76). In the current research, the expression of chemokine *CXCL10* was significantly increased after PRRSV infection in all pigs, but the fold changes in susceptible pigs were higher than resistant pigs at all timepoints and were significantly different at 7 dpi (**Figure 4C(a)** and **Supplementary Figure 2**). In addition, a recent study suggests that *CXCL10* plays a major role in the SARS-COV-2-induced cytokine storm (77). Monocytes are the main sources of cytokine storms, which cause severe inflammatory responses (78). Susceptible pigs had high-level activities in the monocyte related modules. *LRP1* is described as an inflammatory mediator and the down-regulation of *LRP1* in monocytes may promote monocyte recruitment and amplify inflammation (79). The expression levels of *LRP1* at 7 dpi were down-regulated in susceptible pigs while up-regulated in resistant pigs (**Figure 3C** and **Supplementary Figure 2**). Moreover, the overexpression of *MTDH* greatly increased the expression of inflammatory cytokines and was associated with the severity of inflammatory response during viral infection (60, 80, 81). The fold changes of *MTDH* at 7 dpi were significantly higher in susceptible pigs (**Figure 4C(b)** and **Supplementary Figure 2**). These findings suggest that susceptible pigs may suffer more severe inflammatory responses than resistant pigs during early PRRSV infection. Lipids have been reported as the potent signaling molecules to regulate the inflammatory response (82). The majority of DE metabolites between susceptible/resistant pigs at 7 dpi belong to lipids (**Figure 5D**). Although DE metabolites between resistant and susceptible pigs in this study were minor, we noted that myristic acid was significantly higher in serum of susceptible pigs than resistant pigs at both sampling timepoints (**Supplementary Figure 5B**). Myristic acid serum levels are positively correlated with the severity of an inflammatory response (83). Creatinine has the potential to function as an anti-inflammatory agent for both human and animal inflammatory diseases (84). The creatinine levels in resistant pigs were higher than susceptible pigs at 0 dpi and 7 dpi (**Figure 8D**). A previous study also observed a high creatinine level in PRRSV infected fetuses with low viral load compared to fetuses with high viral load (39). Moreover, NK cells response will be inhibited by PRRSV infection, but resistant pigs had higher levels in the modules enriched in NK cells at 4 dpi (**Figure 3B(c)**). Furthermore, the NK cell-associated gene *KLRD1* was up-regulated in resistant pigs but down-regulated in susceptible pigs at 4 dpi (**Figure 3C**). Interestingly, the expression level of *KLRD1* was recently reported as a promising biomarker for predicting human susceptibility to influenza (85). Nevertheless, our data suggest that resistant pigs are capable of initiating faster immune responses to infection with earlier T cell responses whereas susceptible pigs may suffer from excessive inflammatory responses, leading to death.

Overall, PRRSV infection may suppress T cell and B cell activation and promote cytokine storms and inflammatory responses. The majority of DE metabolites were down-regulated in response to PRRSV infection and positively correlated with the expression levels of T cell and B cell marker genes. Some metabolites are involved in the immune response to PRRSV infection and may play important roles in adaptive immunity. Transcriptomic and metabolomic differences after PRRSV infection in susceptible pigs were associated with more severe suppression of T cellular immunity and serious inflammatory responses compared to resistant pigs. T cell-mediated immunity differences may be responsible for the susceptibility/resistance to the PRRSV infection. PRRS vaccines targeting T cell-mediated immunity may provide effective PRRS control in the future. Additionally, previous studies have reported that diet can promote more rapid virus clearance and improve growth performance in PRRSV challenged experiments (86). Further exploration of the impact of DE metabolites on the PRRS resistance by dietary strategies should be carried out in the future.

## Data availability statement

The datasets presented in this study can be found in online repositories. The names of the repository/repositories and accession number(s) can be found below: https://www.ncbi.nlm.nih.gov/, SRP369017.

## Ethics statement

The animal study was reviewed and approved by the Ethical Committee for Animal Experiments at Huazhong Agricultural University, Wuhan, China (Animal experiment approval ID Number: HZAUSW-2017-005).

## Author contributions

XZ and BL conceived the study, designed experiment and revised manuscript. QW performed the data analysis and manuscript writing under the supervision of XZ and BL. YH and XW assisted with the analysis and figure illustration of the data. QS, YS and YW contributed the blood samples and phenotypic values. KG guided the PRRSV artificial infection experiment. JM and ZJ edited the manuscript. All authors contributed to the article and approved the submitted version.

## Funding

This study was supported by the National Natural Science Foundation of China (31930104 and 32172699), the Key Research and Development Program of Hubei Province (2021BBA084), and the Major Project of Hubei Hongshan Laboratory (2021hszd019).

## Acknowledgments

The authors would like to thank Prof. Xiao Shaobo of the Institute of State Key Laboratory of Agricultural Microbiology at Huazhong Agricultural University for providing the PRRSV WUH3 strain and Zhang Qingde of Experimental Animal Centre at Huazhong Agricultural University for providing site for the PRRSV artificial infection experiments.

## Conflict of interest

The authors declare that the research was conducted in the absence of any commercial or financial relationships that could be construed as a potential conflict of interest.

## Publisher’s note

All claims expressed in this article are solely those of the authors and do not necessarily represent those of their affiliated organizations, or those of the publisher, the editors and the reviewers. Any product that may be evaluated in this article, or claim that may be made by its manufacturer, is not guaranteed or endorsed by the publisher.
