# Supplementary material for: Integrated time-series transcriptomic and metabolomic analyses reveal different inflammatory and adaptive immune responses contributing to host resistance to PRRSV
Source: Front Immunol. 2022 Oct 20;13:960709. doi: 10.3389/fimmu.2022.960709 (PMC9631489; doi:10.3389/fimmu.2022.960709)
Supplement: Supplementary file 1 [file DataSheet_1.docx]

Supplementary Material

# Supplementary Figures and Tables

## Supplementary Figures

**A**


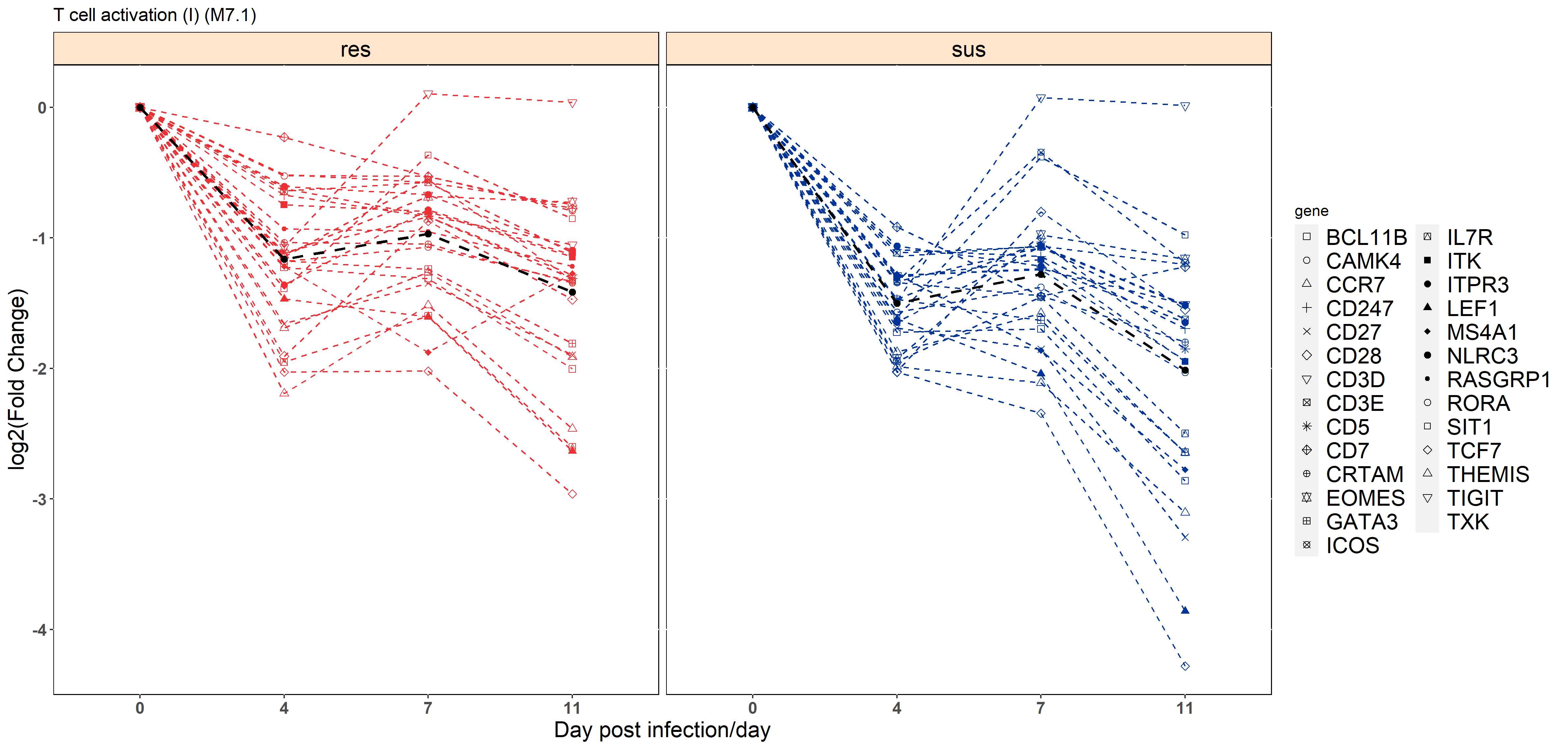


**B**


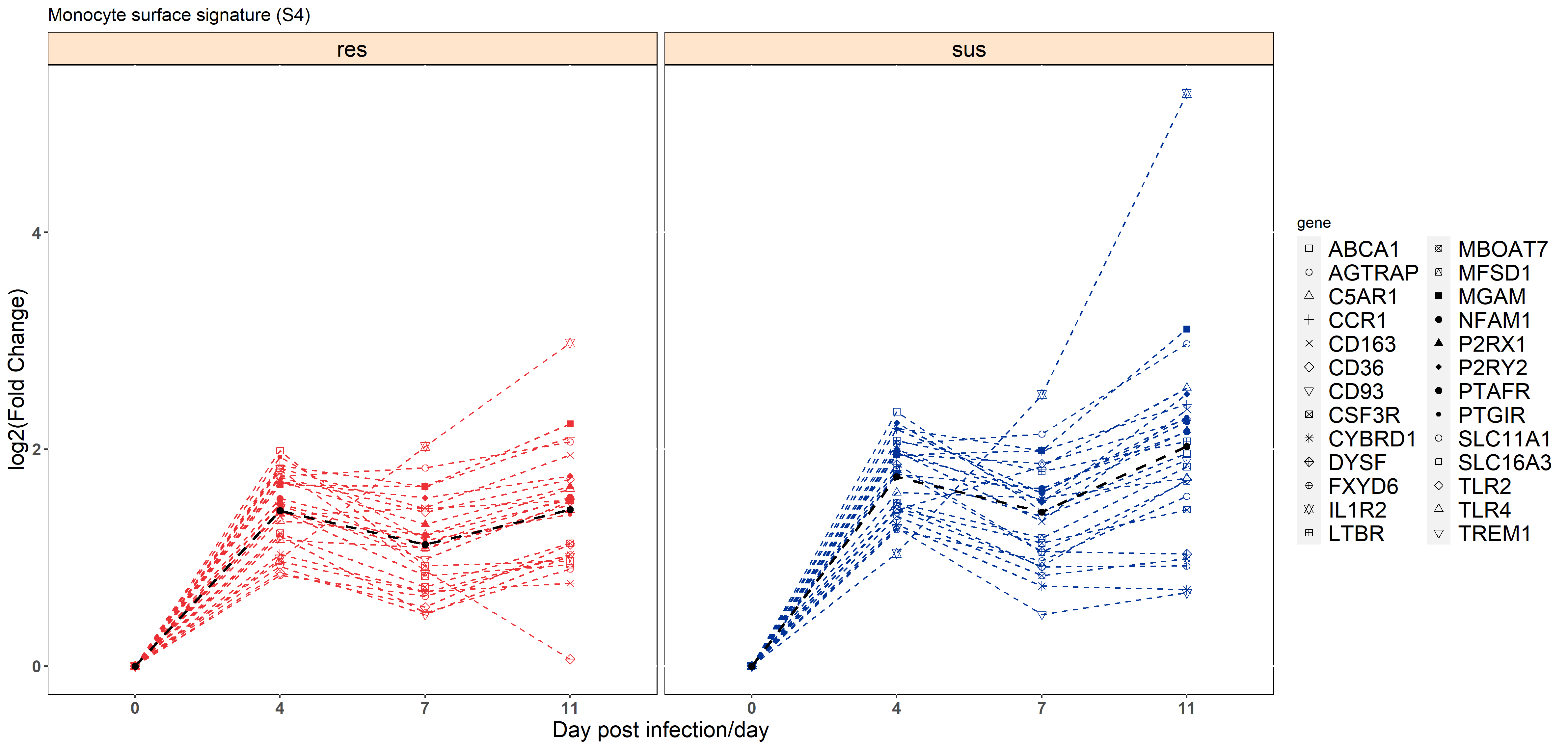


**C**


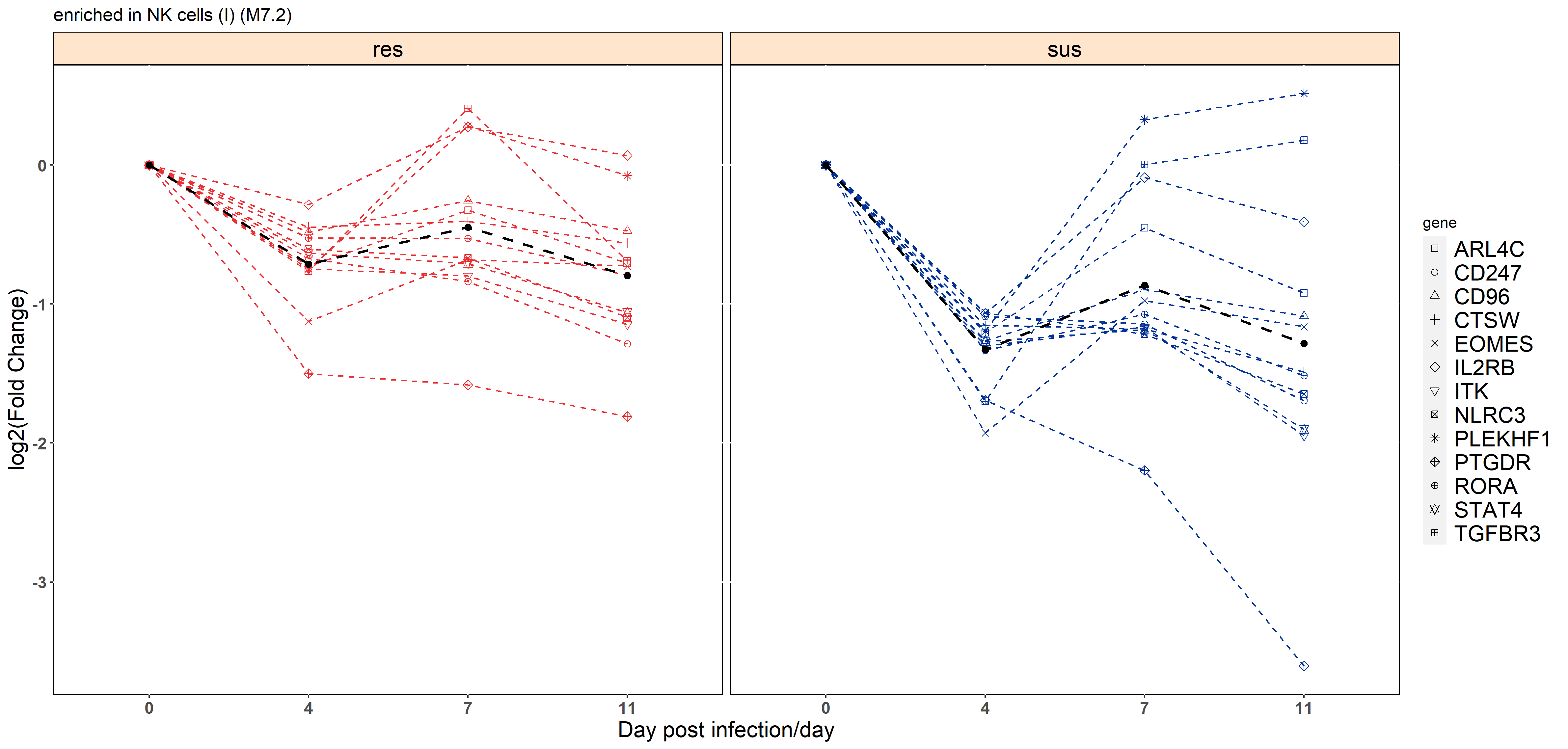


**Supplementary Figure 1.** Temporal expression patterns of genes within modules M7.1 (A), S4 (B) and M7.2 (C) among resistant-(red) and susceptible- individuals (blue); Black line represents the mean fold change of all genes.


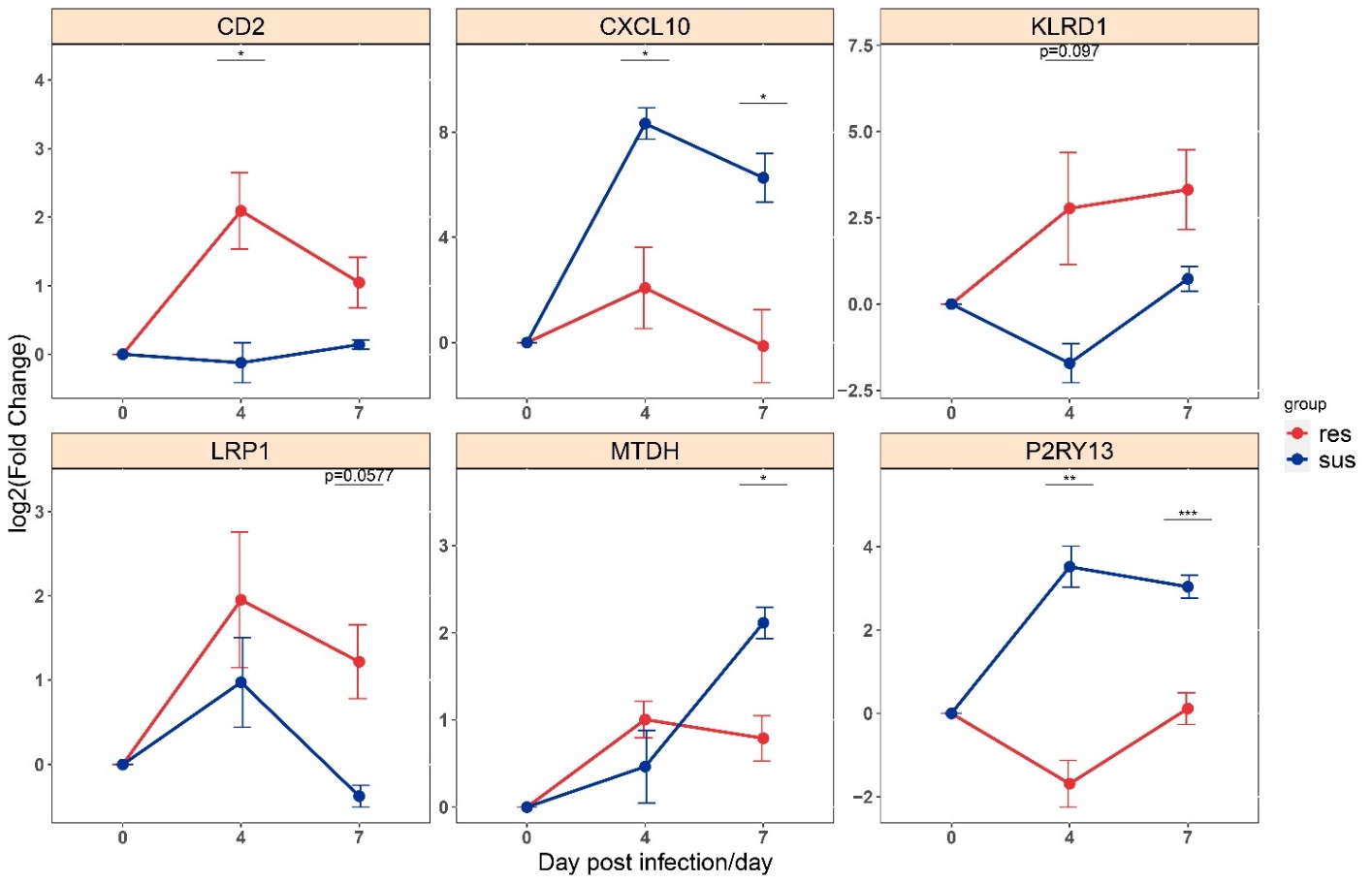


**Supplementary Figure 2.** qPCR validation of DEGs between resistant (red) and susceptible (blue) pigs. Significance levels are shown as *, p-value< 0.05; **, p-value < 0.01; ***, p-value < 0.001.

**
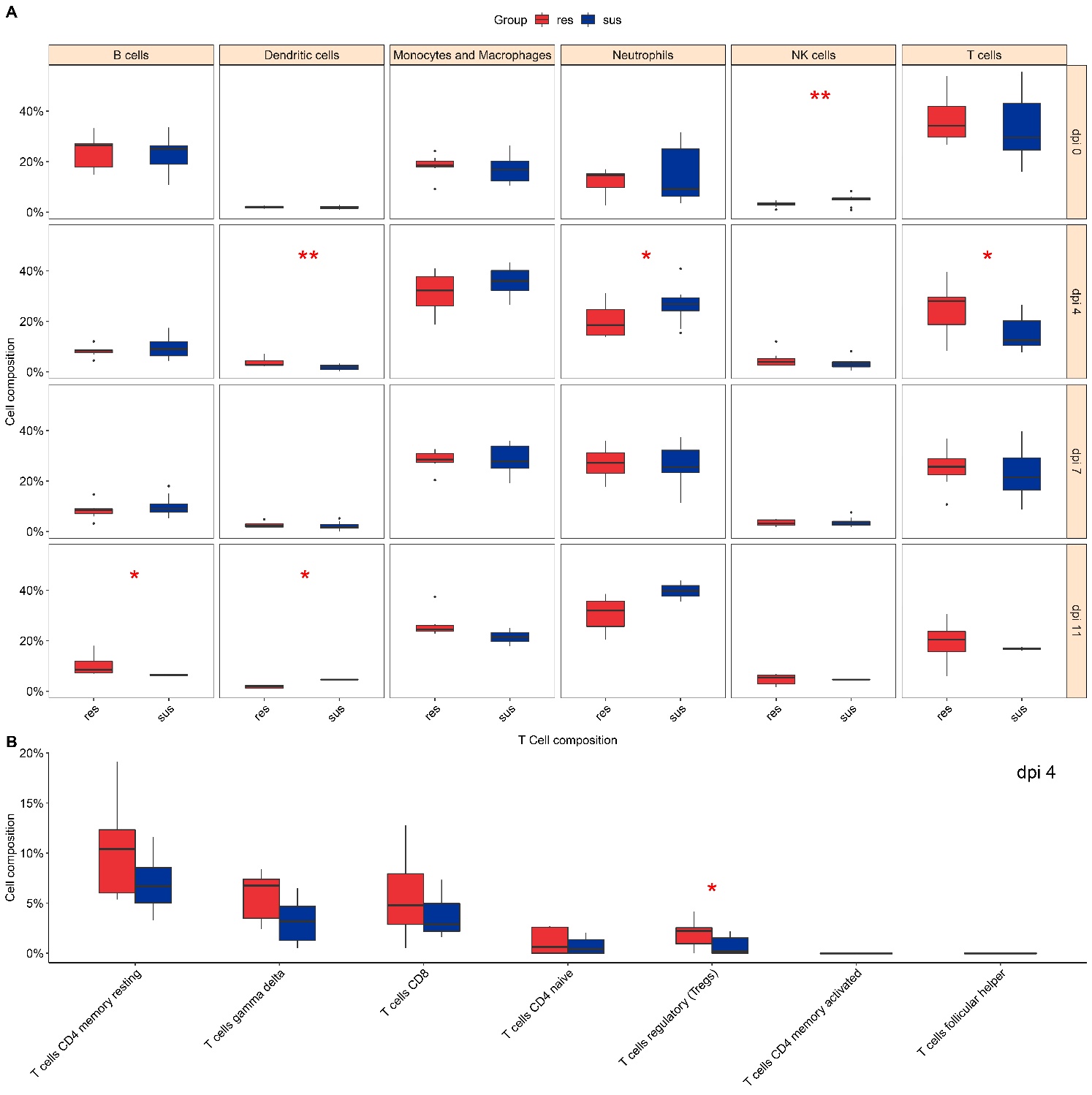
**

**Supplementary Figure 3.** The identification and comparisons of immune cells compositions estimated by CIBERSORT analyses between resistant (red) and susceptible pigs (blue). (A) Immune cell types; (B) T cell subpopulations. Significance levels are shown as *, p-value< 0.10; **, p-value < 0.05; ***, p-value < 0.01.


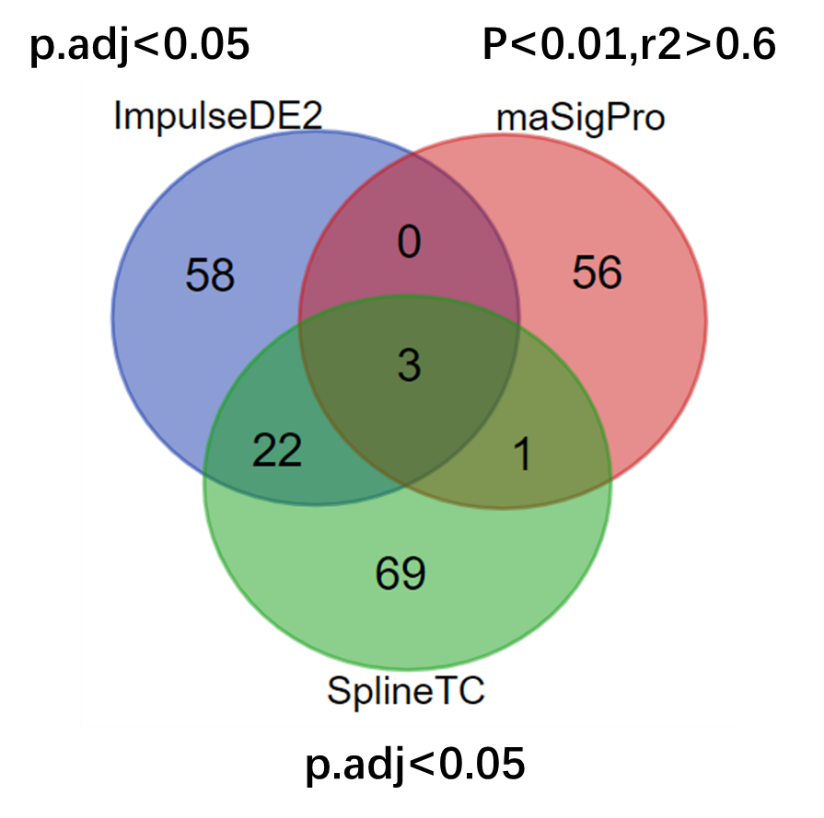


**Supplementary Figure 4.** The differentially expressed genes between resistant- and susceptible- individuals by three time-course analysis methods.


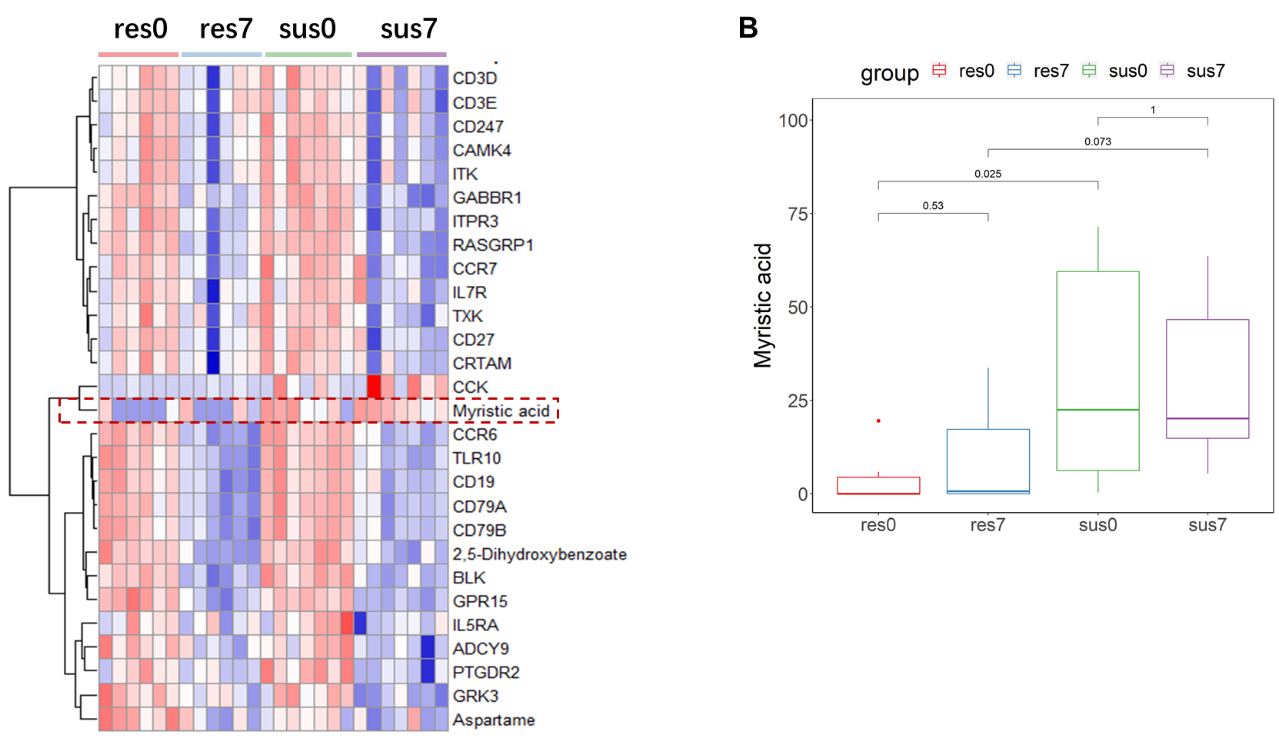


**A**

**Supplementary Figure 5.** (A) Heatmap of differentially expressed genes and metabolites in the network of Figure 9. (B) The expression levels of myristic acid in the four groups (res0, res7, sus0, sus7)


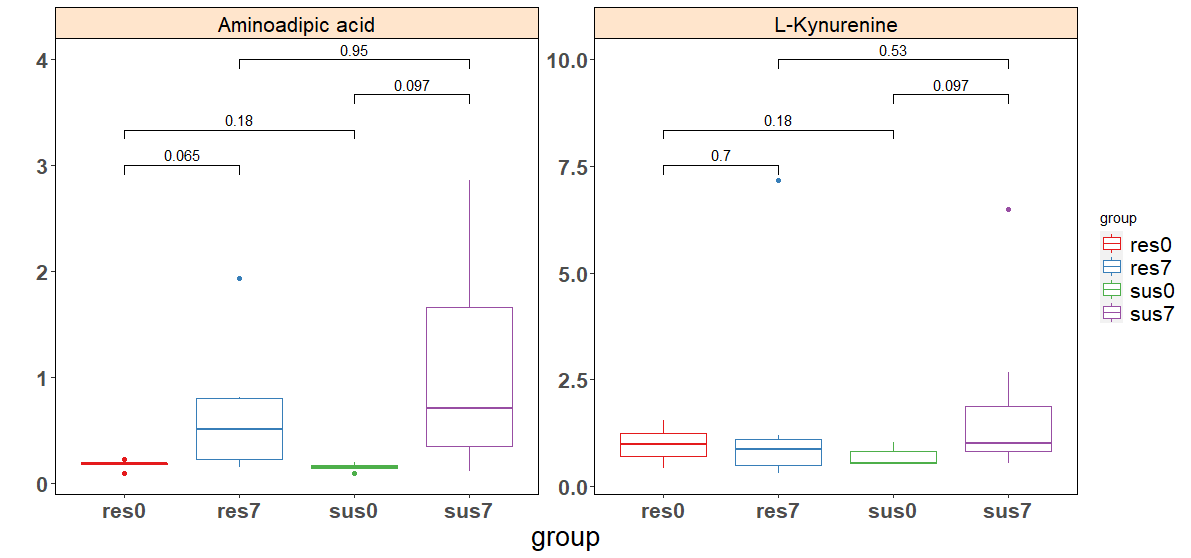


**Supplementary Figure 6.** The expression levels of aminoadipic acid and L-kynurenine in the four groups (res0, res7, sus0, sus7)

## Supplementary Tables

**Supplementary Table 1. Detailed information of samples**

| **ID** | **Gender** | **Age** | **Timepoint to death (dpi)** | **Group** | **Transcriptome** | **Metabolome** |
| --- | --- | --- | --- | --- | --- | --- |
| res1 | male | 84 | - | resistant | ✓ | ✓ |
| res2 | female | 84 | - | resistant | ✓ | ✓ |
| res3 | male | 71 | - | resistant | ✓ | ✓ |
| res4 | female | 64 | - | resistant | ✓ | ✓ |
| res5 | female | 82 | - | resistant | ✓ | ✓ |
| res6 | female | 79 | - | resistant | ✓ | ✓ |
| res7 | female | 65 | - | resistant | ✓ | - |
| sus1 | male | 62 | 8 | susceptible | ✓ | ✓ |
| sus10 | male | 65 | 7 | susceptible | ✓ | - |
| sus2 | female | 92 | 13 | susceptible | ✓ | ✓ |
| sus3 | female | 79 | 12 | susceptible | ✓ | ✓ |
| sus4 | female | 62 | 14 | susceptible | ✓ | ✓ |
| sus5 | male | 100 | 7 | susceptible | ✓ | ✓ |
| sus6 | male | 92 | 9 | susceptible | ✓ | ✓ |
| sus7 | male | 74 | 9 | susceptible | ✓ | ✓ |
| sus8 | male | 61 | 7 | susceptible | ✓ | - |
| sus9 | male | 65 | 8 | susceptible | ✓ | - |

**Supplementary Table 2.** Summary Statistics of Alignment.

| **Sample** | **ID** | **Timepoint (dpi)** | **ReadsNumber** | **Percentage mapped to Genome** |
| --- | --- | --- | --- | --- |
| res1-0 | res1 | 0 | 49081408 | 84.00% |
| res1-4 | res1 | 4 | 57301058 | 85.10% |
| res1-7 | res1 | 7 | 54105781 | 85.20% |
| res1-11 | res1 | 11 | 39021237 | 83.50% |
| res2-0 | res2 | 0 | 37904220 | 82.60% |
| res2-4 | res2 | 4 | 46723065 | 83.80% |
| res2-7 | res2 | 7 | 40509084 | 83.30% |
| res2-11 | res2 | 11 | 37393787 | 82.50% |
| res3-0 | res3 | 0 | 40653068 | 84.00% |
| res3-4 | res3 | 4 | 54156837 | 84.70% |
| res3-7 | res3 | 7 | 42663938 | 85.30% |
| res3-11 | res3 | 11 | 46070922 | 83.60% |
| res4-0 | res4 | 0 | 39700496 | 81.90% |
| res4-4 | res4 | 4 | 35865878 | 83.00% |
| res4-7 | res4 | 7 | 39448894 | 82.60% |
| res4-11 | res4 | 11 | 38280563 | 82.00% |
| res5-0 | res5 | 0 | 43263520 | 85.40% |
| res5-4 | res5 | 4 | 51510078 | 85.80% |
| res5-7 | res5 | 7 | 42439037 | 84.70% |
| res5-11 | res5 | 11 | 35666082 | 85.20% |
| res6-0 | res6 | 0 | 33494958 | 82.30% |
| res6-4 | res6 | 4 | 52303629 | 82.10% |
| res6-7 | res6 | 7 | 43828159 | 81.70% |
| res6-11 | res6 | 11 | 39499613 | 82.50% |
| res7-0 | res7 | 0 | 39576370 | 83.10% |
| res7-4 | res7 | 4 | 47546664 | 84.20% |
| res7-7 | res7 | 7 | 37501708 | 85.50% |
| res7-11 | res7 | 11 | 46019263 | 84.60% |
| sus1-0 | sus1 | 0 | 37532390 | 84.60% |
| sus1-4 | sus1 | 4 | 33736685 | 83.60% |
| sus1-7 | sus1 | 7 | 51077536 | 84.60% |
| sus10-0 | sus10 | 0 | 48053025 | 82.50% |
| sus10-4 | sus10 | 4 | 35113593 | 82.80% |
| sus10-7 | sus10 | 7 | 33157542 | 81.70% |
| sus2-0 | sus2 | 0 | 39656462 | 81.90% |
| sus2-4 | sus2 | 4 | 38722195 | 83.80% |
| sus2-7 | sus2 | 7 | 37138023 | 84.80% |
| sus2-11 | sus2 | 11 | 34312859 | 83.50% |
| sus3-0 | sus3 | 0 | 36136215 | 84.00% |
| sus3-4 | sus3 | 4 | 33869198 | 82.50% |
| sus3-7 | sus3 | 7 | 39056465 | 82.80% |
| sus3-11 | sus3 | 11 | 40587374 | 80.00% |
| sus4-0 | sus4 | 0 | 40341631 | 84.90% |
| sus4-4 | sus4 | 4 | 41885552 | 85.40% |
| sus4-7 | sus4 | 7 | 36461738 | 84.90% |
| sus5-0 | sus5 | 0 | 38622753 | 83.50% |
| sus5-4 | sus5 | 4 | 33393426 | 84.60% |
| sus5-7 | sus5 | 7 | 40603485 | 82.60% |
| sus6-0 | sus6 | 0 | 38968692 | 82.60% |
| sus6-4 | sus6 | 4 | 43076893 | 82.60% |
| sus6-7 | sus6 | 7 | 38933493 | 80.90% |
| sus7-0 | sus7 | 0 | 47395801 | 83.80% |
| sus7-4 | sus7 | 4 | 46216306 | 84.90% |
| sus7-7 | sus7 | 7 | 35196081 | 84.90% |
| sus8-0 | sus8 | 0 | 37673341 | 81.20% |
| sus8-4 | sus8 | 4 | 47335592 | 84.60% |
| sus8-7 | sus8 | 7 | 52304704 | 85.20% |
| sus9-0 | sus9 | 0 | 42855927 | 82.50% |
| sus9-4 | sus9 | 4 | 35457408 | 82.10% |
| sus9-7 | sus9 | 7 | 31975559 | 82.00% |

Total clean reads per sample were aligned to the pig reference genome (Suscrofa 11.1). Samples were labeled in the format of “individual ID-timepoint”, collapsed by “-”.

**Supplementary Table 3.** Primer sequences used for qPCR validation of differentially expressed genes

| **Target gene** | **Forward (F)/reverse (R) primer (5’→3’)** | **Product size (bp)** |
| --- | --- | --- |
| MTDH | F:TCTTCCACCTACTATTTCTACCGA | 215 |
|  | R:ACGTTTCCCGTCTGGCTTT |  |
| P2RY13 | F:TTCCTCATCTCCCTGCCAAAT | 279 |
|  | R:GAAGACAGCCACTACAACAAATAC |  |
| LRP1 | F:GGCCAAGGTCCCAGATGAG | 103 |
|  | R:GGTGTCGGCGAAGTAGATGAA |  |
| KLRD1 | F:CTCCAGGACCCTCCACAGA | 253 |
|  | R:AGCCAGGCATTATGCTCTTC |  |
| CXCL10 | F:GTGCTGTTCTTATTTTCTGCCTTA | 265 |
|  | R:CTTCTCTCTGTGTTCGAGGAGAT |  |
| CD2 | F:GTAATCTCCTGGAGCTGTGC | 186 |
|  | R:CTCACTGGCGTTGTTACTGG |  |

**Supplementary Table 4.** The DEGs overlapped with the PRRSV-related QTLs

| **CHR** | **POS** | **END** | **GENE** | **QTL** |
| --- | --- | --- | --- | --- |
| 1 | 200761004 | 200766056 | CDKN2B | PRRSV antibody titer QTL (9658) |
| 1 | 92447164 | 92483236 | MTO1 | PRRSV antibody titer QTL (9658) |
| 1 | 164657086 | 164735083 | SMAD6 | PRRSV antibody titer QTL (9658) |
| 1 | 104241571 | 104417105 | RAB27B | PRRSV antibody titer QTL (9658) |
| 1 | 183915339 | 184140122 | SAMD4A | PRRSV antibody titer QTL (9658) |
| 4 | 93899026 | 93949398 | LMNA | PRRSV susceptibility QTL (140341) |
| 4 | 98745266 | 98771667 | PRPF3 | PRRSV susceptibility QTL (140341) |
| 4 | 77748810 | 78002009 | ST18 | PRRSV susceptibility QTL (140341) |
| 7 | 43624832 | 43643600 | CENPQ | PRRSV susceptibility QTL (140348) |
| 7 | 53877813 | 53890546 | KLF15 | PRRSV susceptibility QTL (140348) |
| 7 | 55200593 | 55220497 | KIF7 | PRRSV susceptibility QTL (140348) |
| 7 | 43777061 | 43804549 | CRISP3 | PRRSV susceptibility QTL (140348) |
| 9 | 77197535 | 77206526 | TAC1 | PRRSV susceptibility QTL (140353) |
| 14 | 127064397 | 127175227 | SHTN1 | PRRSV antibody titer QTL (194810) |

**Supplementary Table 5.** Detailed information of DE metabolites by one-way ANOVA

| **Feature name** | **f-value** | **p-value** | **LOG10(p)** | **FDR** | **Cluster** |
| --- | --- | --- | --- | --- | --- |
| (+)-7-Isojasmonic acid | 14.393 | 2.08E-05 | 4.6814 | 0.001254 | 5 |
| 12-Hydroxydodecanoic acid | 4.6961 | 0.011088 | 1.9551 | 0.087831 | 5 |
| 2-(Methylamino)benzoic acid | 16.017 | 9.57E-06 | 5.0191 | 0.00072 | 4 |
| 2,5-Dihydroxybenzoate | 17.065 | 5.95E-06 | 5.2252 | 0.000597 | 4 |
| 2-Dehydro-3-deoxy-L-rhamnonate | 5.4673 | 0.005809 | 2.2359 | 0.066055 | 5 |
| 2-Dehydropantoate | 4.7479 | 0.010606 | 1.9745 | 0.087831 | 4 |
| 2-Oxohept-3-enedioate | 5.7104 | 0.004772 | 2.3213 | 0.061682 | 4 |
| 3-Dehydroshikimate | 7.7154 | 0.001058 | 2.9754 | 0.02036 | 5 |
| 3-Hydroxyphenylacetic acid | 10.371 | 0.000188 | 3.7268 | 0.006274 | 2 |
| 4,5-Dihydroorotic acid | 5.6609 | 0.004966 | 2.304 | 0.061682 | 5 |
| 4-Acetamidobutanoic acid | 5.3849 | 0.006214 | 2.2066 | 0.066055 | 5 |
| 4-Pyridoxic acid | 11.016 | 0.000128 | 3.8932 | 0.005499 | 4 |
| 6-Hydroxyhexanoic acid | 5.3891 | 0.006193 | 2.2081 | 0.066055 | 1 |
| 9-OxoODE | 7.0912 | 0.001657 | 2.7808 | 0.029331 | 3 |
| alpha-Ketoisovaleric acid | 4.9037 | 0.009285 | 2.0322 | 0.087339 | 4 |
| Aminoadipic acid | 4.9138 | 0.009206 | 2.0359 | 0.087339 | 3 |
| Aspartame | 5.044 | 0.008248 | 2.0836 | 0.082756 | 1 |
| beta-Alanyl-L-arginine | 6.7504 | 0.002132 | 2.6712 | 0.03565 | 4 |
| Bufotenin | 8.3162 | 0.000699 | 3.1558 | 0.016176 | 2 |
| Butyric acid | 4.719 | 0.010872 | 1.9637 | 0.087831 | 3 |
| Creatinine | 9.1839 | 0.000393 | 3.4051 | 0.01 | 2 |
| Desmosterol | 4.8224 | 0.009951 | 2.0022 | 0.087831 | 3 |
| D-Lysopine | 9.3368 | 0.000357 | 3.4478 | 0.01 | 3 |
| D-Mannose | 5.9175 | 0.004046 | 2.3929 | 0.055361 | 4 |
| gamma-Glutamylcysteine | 6.3063 | 0.002987 | 2.5248 | 0.043042 | 5 |
| Hexadecanedioate | 5.6222 | 0.005123 | 2.2905 | 0.061682 | 2 |
| Hippuric acid | 10.462 | 0.000178 | 3.7507 | 0.006274 | 4 |
| Juvenile hormone III | 6.5854 | 0.002414 | 2.6173 | 0.038239 | 1 |
| L-Alanine | 7.6835 | 0.001082 | 2.9657 | 0.02036 | 5 |
| L-Tryptophan | 5.356 | 0.006364 | 2.1963 | 0.066055 | 4 |
| L-Tyrosine | 4.7191 | 0.010871 | 1.9637 | 0.087831 | 5 |
| Lubiprostone | 4.5204 | 0.012913 | 1.889 | 0.099659 | 1 |
| Myristic acid | 6.2994 | 0.003003 | 2.5225 | 0.043042 | 5 |
| Nicotinuric acid | 11.648 | 8.89E-05 | 4.0509 | 0.004462 | 4 |
| Palmitoyl-L-carnitine | 4.761 | 0.010488 | 1.9793 | 0.087831 | 3 |
| Phosphonoacetate | 20.246 | 1.58E-06 | 5.8014 | 0.000238 | 1 |
| Salicylic acid | 7.7046 | 0.001066 | 2.9721 | 0.02036 | 1 |
| Salicyluric acid | 24.531 | 3.30E-07 | 6.482 | 9.92E-05 | 4 |
| Uracil | 9.1635 | 0.000399 | 3.3994 | 0.01 | 2 |
